# Growth Differentiation Factor 9 (GDF9) Suppresses Follistatin and Follistatin-Like 3 Production in Human Granulosa-Lutein Cells

**DOI:** 10.1371/journal.pone.0022866

**Published:** 2011-08-01

**Authors:** Feng-Tao Shi, Anthony P. Cheung, He-Feng Huang, Peter C. K. Leung

**Affiliations:** 1 Department of Obstetrics and Gynaecology, Child and Family Research Institute, University of British Columbia, Vancouver, British Columbia, Canada; 2 Department of Obstetrics and Gynecology, Zhejiang University School of Medicine, Zhejiang, China; University of Hong Kong, Hong Kong

## Abstract

**Background:**

We have demonstrated that growth differentiation factor 9 (GDF9) enhances activin A-induced inhibin *β*_B_-subunit mRNA levels in human granulosa-lutein (hGL) cells by regulating receptors and key intracellular components of the activin signaling pathway. However, we could not exclude its effects on follistatin (FST) and follistatin-like 3 (FSTL3), well recognized extracellular inhibitors of activin A.

**Methodology:**

hGL cells from women undergoing *in vitro* fertilization (IVF) treatment were cultured with and without siRNA transfection of FST, FSTL3 or GDF9 and then treated with GDF9, activin A, FST, FSTL3 or combinations. FST, FSTL3 and inhibin *β*_B_-subunit mRNA, and FST, FSTL3 and inhibin B protein levels were assessed with real-time RT-PCR and ELISA, respectively. Data were log transformed before ANOVA followed by Tukey's test.

**Principal Findings:**

GDF9 suppressed basal FST and FSTL3 mRNA and protein levels in a time- and dose-dependent manner and inhibited activin A-induced FST and FSTL3 mRNA and protein expression, effects attenuated by BMPR2 extracellular domain (BMPR2 ECD), a GDF9 antagonist. After GDF9 siRNA transfection, basal and activin A-induced FST and FSTL3 mRNA and protein levels increased, but changes were reversed by adding GDF9. Reduced endogenous FST or FSTL3 expression with corresponding siRNA transfection augmented activin A-induced inhibin *β*_B_-subunit mRNA levels as well as inhibin B levels (*P* values all <0.05). Furthermore, the enhancing effects of GDF9 in activin A-induced inhibin *β*_B_-subunit mRNA and inhibin B production were attenuated by adding FST.

**Conclusion:**

GDF9 decreases basal and activin A-induced FST and FSTL3 expression, and this explains, in part, its enhancing effects on activin A-induced inhibin *β*_B_-subunit mRNA expression and inhibin B production in hGL cells.

## Introduction

Inhibin and activin are structurally related dimeric glycoprotein hormones that inhibit and stimulate, respectively, the secretion of FSH by pituitary gonadotrophs. Inhibin A and B are dimers of a common *α*-subunit covalently coupled to *β*_A_- or *β*_B_-subunit, respectively. Activins are dimers of the *β*-subunits: activin A (*β*_A_ *β*_A_), activin B (*β*_B_ *β*_B_), and activin AB (*β*_A_ *β*_B_).

Activin has putative biological actions in a wide variety of tissues such as the pituitary, bone, gonad, liver, and kidney as well as hematopoeitic cells (see Ref. [1], [2]). Activin signaling is regulated by a number of extracellular and intracellular mechanisms [3]. Among the extracellular regulators are some members of the follistatin (FST)-related protein family. This family consists of a large group of proteins which contain a highly conserved module of cysteine-rich sequence termed the follistatin domain and includes follistatin [4], follistatin-like (FSTL) proteins such as FSTL3 [5], and agrin [6]. Apart from follistatin and FSTL3 {formerly known as FST-related protein (FSRP) and FST-related gene (FLRG)} which are major regulators of activin action, most proteins in this family do not have this follistatin-like activity. Like follistatin, FSTL3 binds activin with high affinity and prevents activin from binding to its receptors, and hence, neutralizes activin biological activities [7], [8].

GDF9 was first discovered in 1993 and identified as a member of TGF*β* superfamily [9]. In most mammalian species, GDF9 is expressed predominantly in the growing oocytes from the primary follicle stage onwards until fertilization and plays important roles during follicle development [10]–[15]. Although GDF9 and activin A bind to different receptors, both activate the Smad signaling pathway (Smad 2/3/4/7) downstream. We have recently reported [16], [17] that exogenous and endogenous GDF9 can significantly enhance activin A-induced inhibin *β*_B_-subunit mRNA levels by inducing activin receptors (ACVR2B/1B) and Smad2/3 but reducing Smad7 (an inhibitory Smad) mRNA levels in human granulosa-lutein (hGL) cells. We have also confirmed GDF9 expression in hGL cells [17]. However, its effects on extracellular regulators of activin such as FST and FSTL3 remain unknown.

Activin A can promote FST production in undifferentiated and partially differentiated rat granulosa cells, but suppress FST production in fully differentiated granulosa cells [18]. Activin A can cause a 1.8-fold rise in FST release in rat anterior pituitary cells, suggesting an autocrine/paracrine role of activin and FST in the pituitary [19]. Activin A can also increase FST and FSTL3 mRNA and protein levels in the human hepatoma cell line HepG2 [20] but whether the same occurs in human granulosa cells is unknown. The objectives of the present work were to examine the effects of GDF9 on FST and FSTL3 expression, with and without activin A treatment, as potential mechanisms on its enhancing action on activin A-induced inhibin *β*_B_-subunit mRNA expression and inhibin B production in hGL cells. Because FST has two isoforms, FST288 and FST315, we also compared the effects of these isoforms on the actions of activin A.

## Materials and Methods

Firstly, we compared FST and FSTL3 mRNA in hGL cells and protein in culture media with and without GDF9 treatment in time- and dose-dependent experiments. Secondly, we explored the effects of GDF9 on activin A-induced FST and FSTL3 mRNA and protein levels. Thirdly, we transfected hGL cells with GDF9 siRNA to assess changes in basal and activin A-induced FST and FSTL3 levels. Fourthly, we compared inhibin *β*_B_-subunit mRNA levels after activin A treatment with and without FST or FSTL3 siRNA. Finally, to further evaluate if the enhancing effect of GDF9 on activin A-induced inhibin *β*_B_-subunit mRNA is related to FST or FSTL3 expression, we measured these changes in activin A-treated hGL cells (with and without GDF9) at different doses of FST or FSTL3.

### Preparation of hGL cells

The study was approved by the Research Ethics Board of the University of British Columbia. hGL cells were obtained from women undergoing *in vitro* fertilization treatment, and written informed consent was obtained from all participants involved in this study. For each patient, cells from multiple follicles and consequently follicular fluid were pooled respectively. Granulosa cells from each patient were extracted as described previously [16]. 2×10^5^ viable cells were seeded per well in 12-well culture plates and cultured in DMEM/F-12 (Sigma Chemical Co., St. Louis, MO) supplemented with 10% fetal bovine serum (FBS; HyClone Laboratories, Logan, UT), 100 U/ml penicillin (GIBCO BRL Life Technologies, Grand Island, NY), 100 *µ*g/ml streptomycin sulphate (GIBCO) and 1 × GlutaMAX™ (GIBCO) in a humidified atmosphere of 5% CO_2_-95% air at 37 C for 48 h.

### Activin A, GDF9, FST and FSTL3 experiments

After preculture of hGL cells for 48 h, the above medium containing 0.5% FBS (“low-serum media”) instead of 10% FBS was added to each well and the cell culture was now designated as “Time 0 h” for all subsequent experiments described below. The experimental conditions, and the rationale for selecting the dose of 25 ng/ml of recombinant human activin A (Sigma) and 100 ng/ml of recombinant human GDF9 (Peprotech Inc., Rocky Hill, NJ) for our experiments were detailed in our recent publications [16], [17]. For time-dependent experiments, cells were treated with the dose of 100 ng/ml of GDF9 for 12, 24 and 48 h. Cells were stimulated with 1–200 ng/ml of GDF9 for 24 h in dose-response studies. For experiments with both GDF9 and activin A, cells were preincubated with 100 ng/ml of GDF9 in low-serum media for 24 h before stimulation with 25 ng/ml of activin A. In neutralization experiments to render GDF9 inactive, 2 *µ*g/ml of recombinant extracellular domain (ECD) fused to the Fc region of human IgG (receptor-ECD/Fc chimera) of human BMP receptor-2 (BMPR2 ECD, a GDF9 antagonist; R&D Systems, Minneapolis, MN) and 100 ng/ml of GDF9 were preincubated in low-serum media for 30 min before adding to cultured hGL cells. To test the effects of FST or FSTL3 on activin A-induced inhibin *β*_B_-subunit mRNA expression, different doses of each of the two recombinant human FST isoforms, 288-amino acid FST (FST288; Peprotech) and 315-amino acid FST (FST315; R&D Systems), or FSTL3 (R&D Systems) were preincubated with 25 ng/ml activin A at 37 C for 1 h in PBS containing 0.1% BSA before adding to hGL cells which were then cultured for 24 h.

### Knockdown analysis for human GDF9, FST or FSTL3

We performed transient knockdown assays with 80 nM of GDF9, FST or FSTL3 siRNA using non-targeting siRNA (ON-TARGETplus SMARTpool; Dharmacon Research, Inc., Lafayette, CO) as control. After pre-culture of hGL cells for 48 h, the media were replaced with fresh antibiotics-free culture media, and non-targeting siRNA (“Control siRNA”), GDF9 siRNA, FST siRNA or FSTL3 siRNA was then added with Lipofectamine RNAiMAX (Invitrogen, Carlsbad, CA) to the culture media. The cell culture, immediately after adding transfection reagents, was now designated as “Time 0 h” for all subsequent experiments described below. After 24 h (“Time 24 h”), the spent media were replaced with fresh antibiotic-free culture media to remove the transfection reagents and the hGL cells were cultured for another 24 hours (“Time 48 h”). The spent media were then replaced with low-serum media and hGL cells were further cultured for 24 h (“Time 72 h”). mRNA levels of FST or FSTL3 were then quantified with real-time PCR at 48 and 72 h after adding the transfection reagents. Corresponding protein levels were quantified with ELISA at 72 h after siRNA transfection. In separate experiments, the spent media at “Time 48 h” were replaced with low-serum media and hGL cells were incubated with and without 100 ng/ml GDF9 for one more day (“Time 72 h”), and FST or FSTL3 mRNA in cells and protein levels in culture media were quantified with real-time PCR and ELISA respectively.

### RNA extraction and Real-time RT-PCR

At the end of the treatment period, RNA was extracted from hGL cells using TRIzol (Invitrogen). The experimental procedures for RNA extraction, first-strand cDNA synthesis and SYBR Green real-time PCR have been detailed in our recent publication [16]. The primers used for SYBR Green real-time PCR were designed using the Primer Express Software (Applied Biosystems, Foster City, CA) (Table 1) and tested with the intron spanning assay. Expression levels of the interested genes were quantified and normalized to those of the human housekeeping gene, GAPDH. Changes after treatments were recorded as fold differences from values in untreated controls at each time point as appropriate.

**Table 1:** Nucleotide sequences of primers used for quantitative real-time PCR.

Gene	Forward primer sequence	Reverse primer sequence
FST	5′-TGCTCTGCCAGTTCATGG-3′	5′-CTTGACGGAGCCAGCAGT-3′
FSTL3	5′-CTACATCTCCTCGTGCCACA-3′	5′-TCTTCTGCAGACTCACCACCT-3′
Inhibin *β*_B_-subunit	5′-ATCAGCTTCGCCGAGACA-3′	5′-GCCTTCGTTGGAGATGAAGA-3′
GAPDH	5′-ATGGAAATCCCATCACCATCTT-3′	5′-CGCCCCACTTGATTTTGG-3′

### FST, FSTL3 and inhibin B assays

After treatment, culture media were collected and stored frozen until assay with specific ELISA kits (FST, Peprotech; FSTL3, R&D Systems; inhibin B, Diagnostic Systems Laboratories, Webster, TX); corresponding hGL cells were lysed with lysis buffer, and total cellular protein content determined as described earlier. The lowest limits of detection for FST, FSTL3 and inhibin B were 23, 312.5 and 7 pg/ml, respectively. The corresponding intraassay coefficients of variation were 2.36, 1.92 and 3.5%; the interassay coefficients of variation were 2.74, 2.12 and 6.20%. All hormone measurements were performed in duplicate. Secreted hormone levels were normalized to total cellular protein content.

### Statistical analysis

Results were the means ± SEM from at least four sets of replicated experiments (each from a separate patient), and in each set, measurements were made in triplicate for real-time PCR or duplicate for ELISA. Data were log transformed and analyzed by one-way ANOVA followed by Tukey's multiple comparison tests if the overall P values were significant at *P*<0.05 using PRISM software (GraphPad Software, Inc., San Diego, CA).

## Results

### Effects of GDF9 on FST and FSTL3 mRNA and protein levels

**Figure 1:**
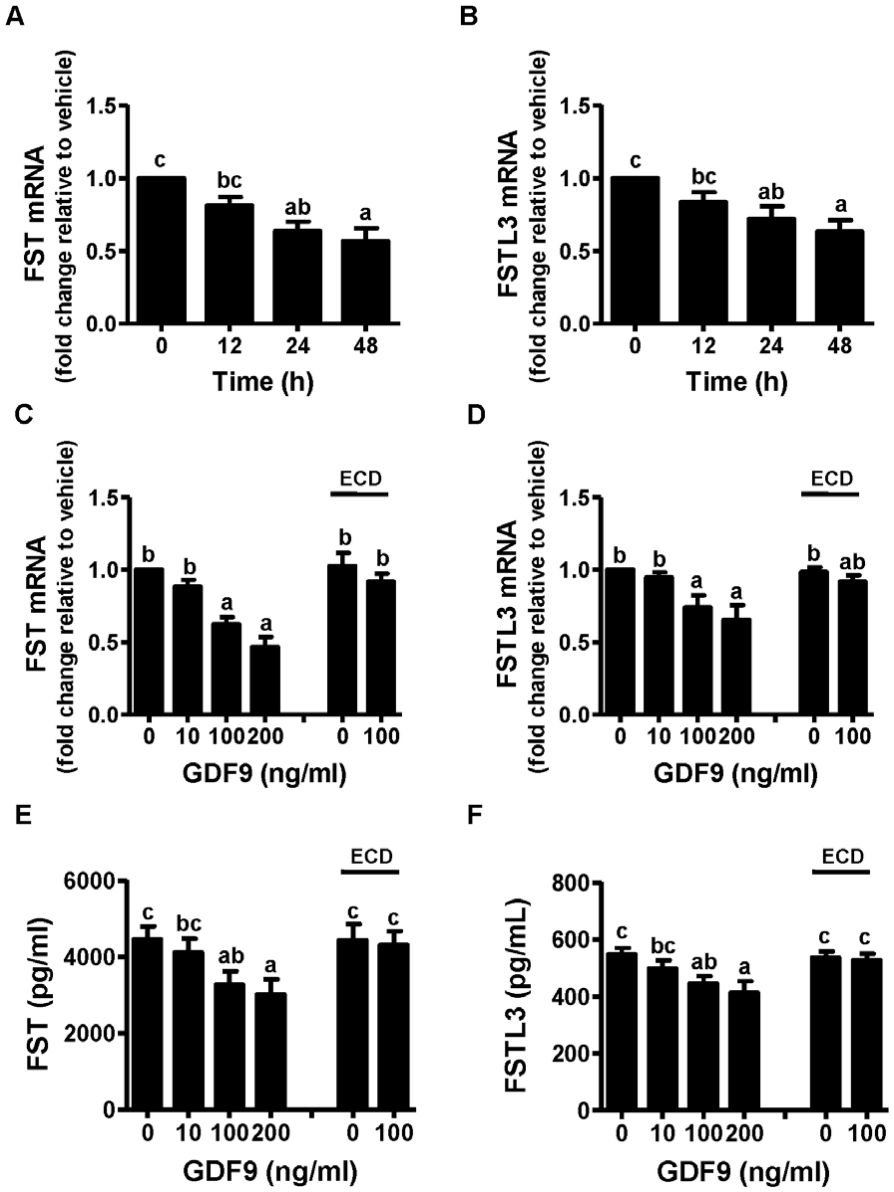
Effects of GDF9 on FST and FSTL3 mRNA and protein levels. After 48 h preculture, the culture media were replaced with low-serum media (0.5% FBS); hGL cells were then treated with 100 ng/ml GDF9 for 12 h (“Time 12 h”), 24 h (“Time 24 h”) and 48 h (“Time 48 h”) in time-dependent experiments (A and B), or with different doses of GDF9 for 24 h in dose-dependent experiments (C, D, E and F). In neutralization experiments to render GDF9 inactive, 2 *µ*g/ml of BMPR2 ECD (“ECD”) and 100 ng/ml of GDF9 were preincubated in low-serum media for 30 min before adding to cultured hGL cells. FST and FSTL3 mRNA levels (A, B, C and D) in hGL cells and protein concentrations (E and F) in culture media were assessed by real-time PCR and ELISA, respectively. Results were the means ± SEM from at least four sets of experiments (each from a separate patient), and in each set, measurements were made in triplicate for real-time PCR or duplicate for ELISA. *Means without a common letter* are significantly different (*P*<0.05).

GDF9 significantly decreased FST and FSTL3 mRNA levels in a time-dependent manner with maximum effects at 48 h (Figure 1, A and B). GDF9 also reduced mRNA levels of FST and FSTL3 in a dose-dependent manner which reached statistical significance at both the 100 and 200 ng/ml doses (*P* values all <0.01) (Figure 1, C and D). BMPR2 is the type 2 receptor for GDF9 and the ECD of BMPR2 is a well known GDF9 antagonist [16], [17], [21]–[23]. When 100 ng/ml of GDF9 was preincubated with BMPR2 ECD for 30 min before adding to the cell culture, the inhibitory effects of GDF9 on FST and FSTL3 mRNA levels were attenuated (Figure 1, C and D). Correspondingly, GDF9 decreased FST and FSTL3 protein levels in a dose-dependent manner and reached statistical significance at the 100 ng/ml dose (Figure 1, E and F; *P* values all <0.05); as a result, no significant decreases were observed when GDF9 was first neutralized with BMPR2 ECD (Figure 1E and F). As expected, there were no significant changes in FST and FSTL3 mRNA levels relative to controls when BMPR2 ECD alone was added. Basal protein levels of FST in culture media were higher than those of FSTL3 (Figure 1, E and F, 4450 *vs*. 548 pg/ml); correspondingly, the percentage decreases in FST protein levels following GDF9 treatment were greater than those of FSTL3 (32.4% *vs.* 24.8% at the GDF9 dose of 200 ng/ml; *P* value <0.001).

### Effects of GDF9 on activin A-induced FST and FSTL3 mRNA and protein levels

**Figure 2:**
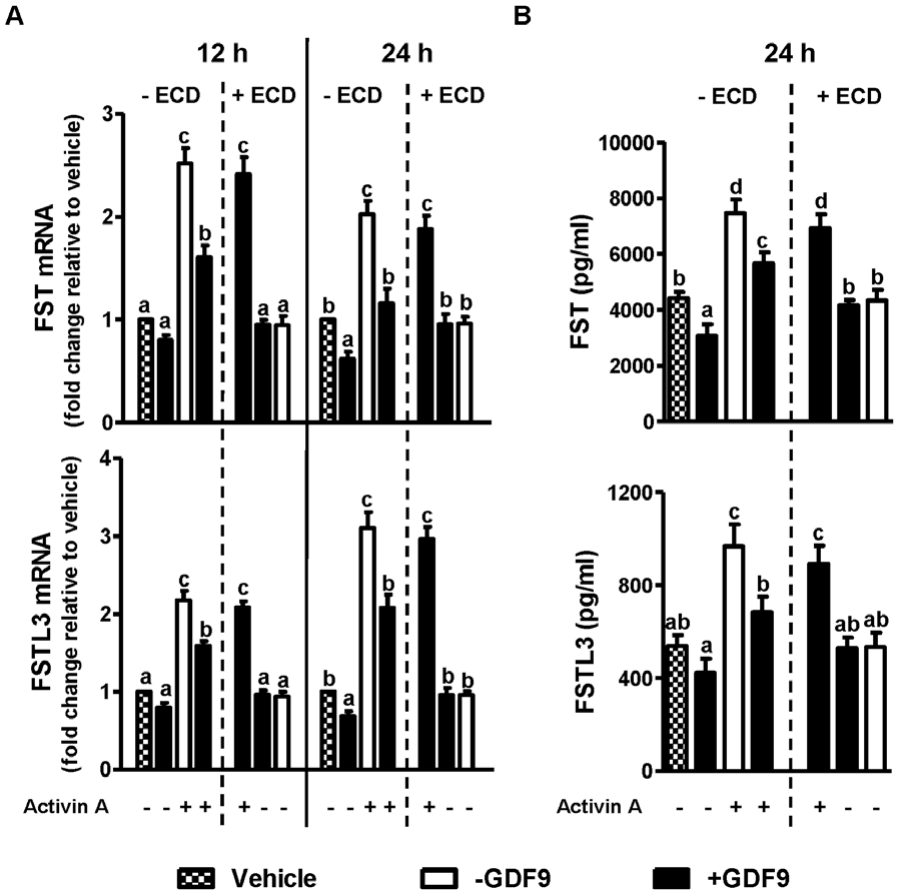
GDF9 reversed activin A-induced FST and FSTL3 expression, effects attenuated by BMPR2 ECD. After 48 h preculture, hGL cells were incubated in low-serum media (0.5% FBS) with and without 100 ng/ml of GDF9 for another 24 h before stimulation with 25 ng/ml of activin A for 12 h (“Time 12 h”) and 24 h (“Time 24 h”). The neutralization experiments with BMPR2 ECD (“ECD”) and GDF9 were as described in Figure 1. FST and FSTL3 mRNA levels (A) in hGL cells and protein concentrations (B) in culture media were assessed by real-time PCR and ELISA, respectively. Results were the means ± SEM from at least four sets of experiments (each from a separate patient), and in each set, measurements were made in triplicate for real-time PCR or duplicate for ELISA. At each time point, *means without a common letter* are significantly different (*P*<0.05).

Activin A increased both FST and FSTL3 mRNA levels (Figure 2A; *P* values all <0.001). In contrast, GDF9 suppressed basal and activin A-induced FST and FSTL3 mRNA levels, effects that were attenuated by BMPR2 ECD (Figure 2A; *P* values all <0.05). As noted earlier, BMPR2 ECD alone had no effects on FST and FSTL3 mRNA levels. Changes in FST and FSTL3 protein levels in culture media followed an identical pattern to changes in mRNA levels (Figure 2B). However, FST mRNA levels peaked at 12 h while those of FSTL3 peaked at 24 h in response to activin A or activin A with GDF9 (Figure 2A).

### Effects of GDF9-targeting siRNA transfection on activin A-induced FST and FSTL3 mRNA and protein levels

**Figure 3:**
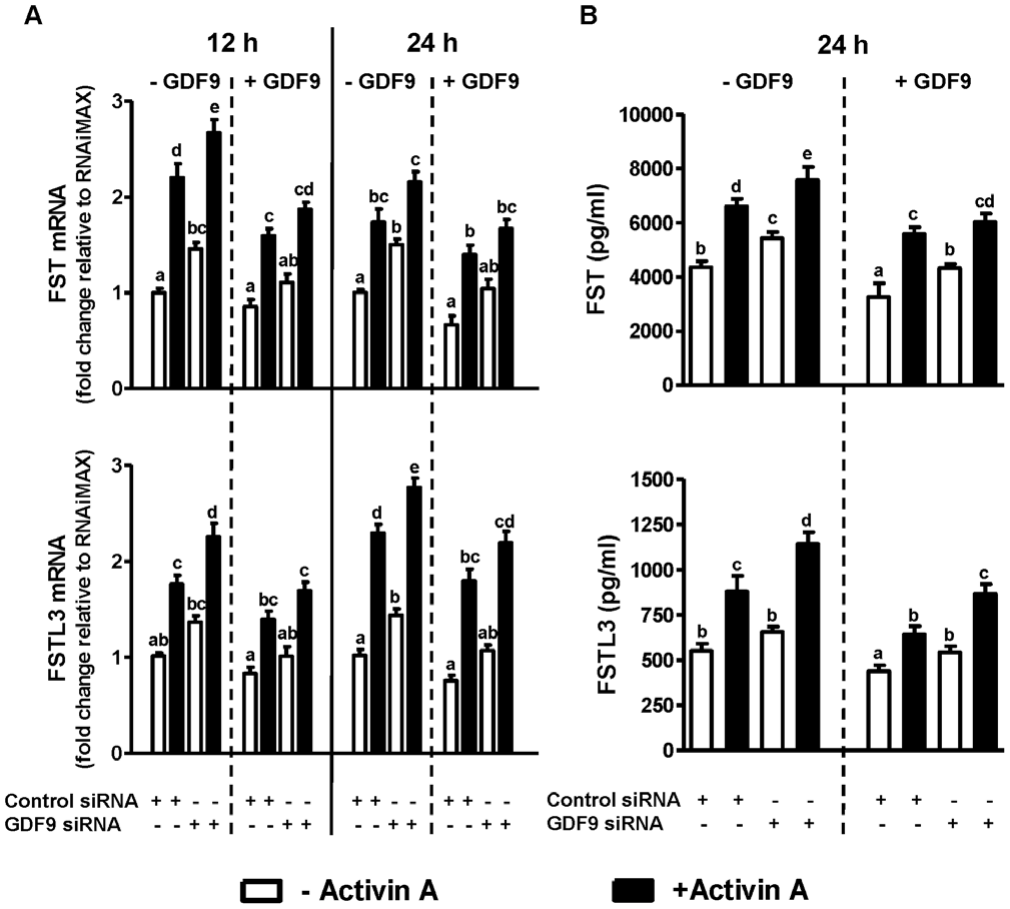
GDF9-targeting siRNA increased activin A-induced FST and FSTL3 expression, effects reversed by GDF9 treatment. After preculture for 48 h, hGL cells were transfected with 80 nM GDF9 targeting siRNA for 48 h. The culture media were then replaced by low-serum media (0.5% FBS), and hGL cells were cultured with and without 100 ng/ml GDF9 for another 24 h period before stimulation with 25 ng/ml of activin A for 12 h (“Time 12 h”) and 24 h (“Time 24 h”). FST and FSTL3 mRNA levels (A) in hGL cells and protein concentrations (B) in culture media were assessed by real-time PCR and ELISA, respectively. Results were the means ± SEM from at least four sets of experiments (each from a separate patient), and in each set, measurements were made in triplicate for real-time PCR or duplicate for ELISA. At each time point, *means without a common letter* are significantly different (*P*<0.05).

When endogenous GDF9 levels decreased following GDF9 siRNA transfection (75% suppression of GDF9 mRNA level at 48 h, see Figure 1B of Ref. [17] for details), there were significant increases in mRNA levels of FST and FSTL3 at 60 h and 72 h, corresponding to “Time 12 h” and “Time 24 h” after activin A treatment respectively as shown in Figure 3A (*P* values all <0.001), and corresponding proteins levels (Figure 3B; *P* values all <0.001). Furthermore, these effects of GDF9 siRNA were attenuated at 36 and 48 h after 100 ng/ml GDF9 was added to the culture (corresponding to “Time 12 h” and “Time 24 h” after activin A treatment in Figure 3). As a comparison, transfection with control siRNA showed no changes relative to transfection reagent alone (“RNAiMAX”). GDF9 siRNA transfection increased both basal and activin A-induced FST mRNA levels at 12 h (*P* values all <0.05) and 24 h in hGL cells (Figure 3A, *upper* panel), and basal and activin A-induced FSTL3 mRNA at 12 and 24 h (Figure 3A, *lower* panel), effects that were attenuated when cells were pre-treated with 100 ng/ml GDF9 which reached statistical significance at 12 h after activin A treatment for FST mRNA (*P* value <0.05); and at both 12 h and 24 h after activin A treatment for FSTL3 mRNA (*P* values all <0.05 for both time points). Corresponding changes in protein concentrations of FST and FSTL3 in the culture media showed a similar pattern to mRNA levels (Figure 3B).

### FST- or FSTL3-targeting siRNA enhanced activin A-induced inhibin β_B_-subunit mRNA and inhibin B protein levels

**Figure 4:**
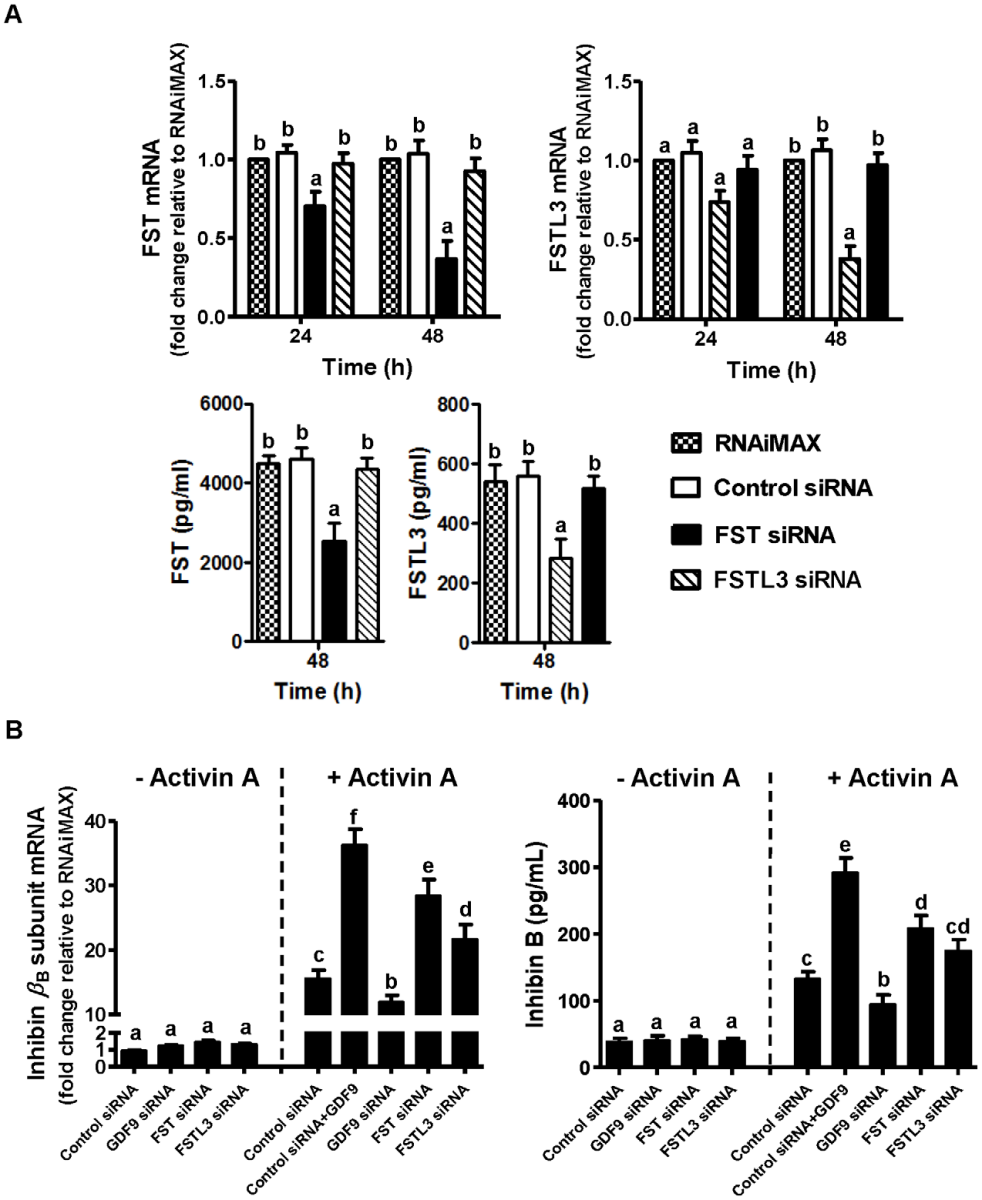
GDF9-targeting siRNA attenuated while FST/FSTL3-targeting siRNA enhanced activin A-induced inhibin *β*_B_-subunit and inhibin B expression. A: After preculture for 48 h, hGL cells were transfected with 80 nM of non targeting siRNA (“Control siRNA”) and FST- or FSTL3-targeting siRNA for 24 h (“Time 24 h”) and 48 h (“Time 48 h”). FST and FSTL3 mRNA levels in hGL cells (*upper* panel) and protein concentrations in culture media (*lower* panel) were assessed by real-time PCR and ELISA, respectively. Results were the means ± SEM from at least four sets of experiments (each from a separate patient), and in each set, measurements were made in triplicate for real-time PCR or duplicate for ELISA. At each time point, *means without a common letter* are significantly different (*P*<0.05). B: After preculture for 48 h, hGL cells were transfected with 80 nM of control siRNA and GDF9-, FST- or FSTL3-targeting siRNA for 48 h. The culture media were then replaced by low-serum media (0.5% FBS), and hGL cells were cultured with and without 100 ng/ml GDF9 for another 24 h before treatment with 25 ng/ml activin A for 24 h. Inhibin *β*_B_-subunit mRNA levels in hGL cells and inhibin B protein levels in culture media were measured by real-time PCR and ELISA respectively. Results were the means ± SEM from at least four sets of experiments (each from a separate patient), and in each set, measurements were made in triplicate for real-time PCR and duplicate for ELISA. *Means without a common letter* are significantly different (*P*<0.05).

As shown in Figure 4A, FST siRNA was specific for FST and had no effects on FSTL3 mRNA and protein levels; similarly, FSTL3 siRNA was specific for FSTL3 and had no effects on FST mRNA and protein levels. As expected, control siRNA had no effect on the basal inhibin *β*_B_-subunit mRNA levels; in addition, in the absence of activin A treatment, GDF9-, FST- or FSTL3-targeting siRNA had no effect on the basal inhibin *β*_B_-subunit mRNA levels (Figure 4B, *left panel*). In contrast, in the presence of activin A and consistent with our previous study [17], GDF9 treatment increased while GDF9 siRNA transfection decreased inhibin *β*_B_-subunit mRNA levels (Figure 4B, *left panel*; *P* values all <0.05). With reduced endogenous FST or FSTL3 expression after targeting siRNA transfection (Figure 4A; *P* values all <0.001 at 48 h), activin A-induced inhibin *β*_B_-subunit mRNA levels increased from 15.7 fold for control siRNA to 27.7 fold for FST siRNA and 21 fold for FSTL3 siRNA relative to RNAiMAX (Figure 4B, *left panel*; *P* values all <0.05). Corresponding changes in inhibin B productions were similarly observed (Figure 4B, *right panel*).

### FST or FSTL3 reversed the effects of GDF9 on activin A-induced inhibin β_B_-subunit mRNA and inhibin B protein levels

**Figure 5:**
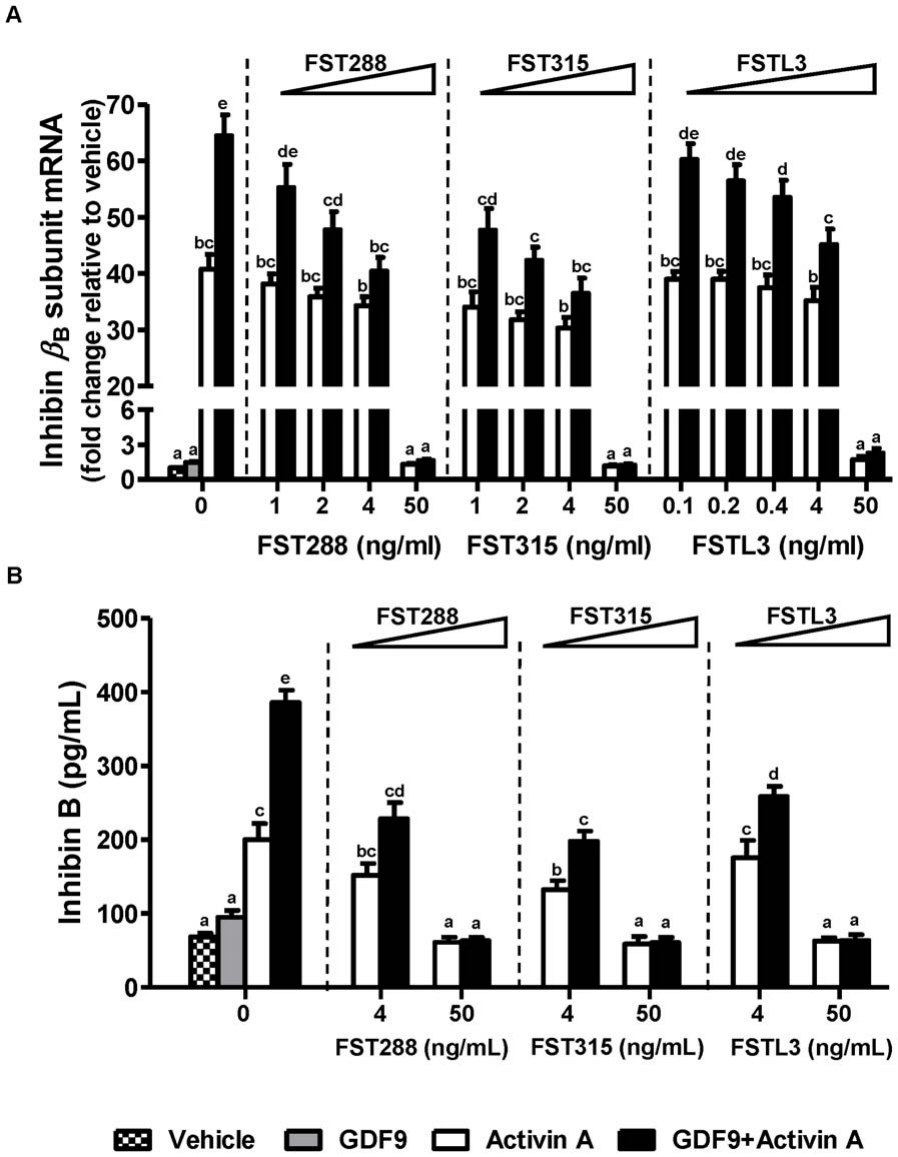
GDF9 pretreatment enhanced activin A-induced inhibin *β*_B_-subunit and inhibin B expression, effects attenuated by FST/FSTL3. After 48 h preculture, the culture media were replaced by low-serum media (0.5% FBS), and hGL cells were cultured with and without 100 ng/ml GDF9 for another 24 h in low-serum media. Different dose of FST288, FST315 or FSTL3 were preincubated with 25 ng activin A at 37 C for 1 h in 50 *µ*l PBS containing 0.1% BSA before adding to the cells and cultured for another 24 h. Inhibin *β*_B_-subunit mRNA levels in hGL cells (A) and inhibin B protein levels in culture media (B) were measured by real-time PCR and ELISA respectively. Results were the means ± SEM from at least four sets of experiments (each from a separate patient), and in each set, measurements were made in triplicate for real-time PCR and duplicate for ELISA. *Means without a common letter* are significantly different (*P*<0.05).

To determine if GDF9 enhanced activin A-induced inhibin *β*_B_-subunit mRNA levels by reducing FST or FSTL3 expression, we compared these changes in the presence of different doses of FST288, FST315 or FSTL3. We chose FST288 or FST315 doses of 1, 2, 4 and 50 ng/ml and FSTL3 doses of 0.1, 0.2, 0.4, 4 and 50 ng/ml based on changes in FST and FSTL3 protein levels following GDF9 treatment (Figure 1, E and F) and that FST or FSTL3 binds to activin in a 2∶1 molar ratio [24]–[27]. Increasing doses of FST288 (1–4 ng/ml), FST315 (1–4 ng/ml) or FSTL3 (0.1–0.4 ng/ml) attenuated activin A-induced inhibin *β*_B_-subunit mRNA levels with levels completely suppressed to those of activin A treatment alone at the saturated dose of 50 ng/ml for both FST isoforms and FSTL3 (Figure 5A). In the presence of GDF9, inhibin *β*_B_-subunit mRNA levels decreased in a dose-dependent manner for FST288, FST315, or FSTL3 (Figure 5A). Corresponding changes in inhibin B production were likewise observed (Figure 5B). While the suppressive effects of FST315 on activin A appeared to be greater than those of FST288 at equivalent doses below the saturated dose of 50 ng/ml, differences did not reach statistical significance (Figure 5).

## Discussion

We have recently reported that exogenous and endogenous GDF9 enhances activin A-induced expression of inhibin *β*_B_-subunit mRNA and inhibin B in hGL cells through modulation of activin receptors and key components of the intracellular signaling pathway [16], [17]. Using the same hGL cell culture system in our current study, we have demonstrated for the first time that GDF9 can decrease not only basal but also activin A-induced mRNA and protein levels of FST and FSTL3, known extracellular inhibitors of activin [7], [28]. These actions of GDF9 were supported by our experiments when GDF9 effects were neutralized by BMPR2 ECD or when endogenous GDF9 levels were reduced by targeting siRNA transfection. We therefore hypothesize that GDF9 decreases FST or FSTL3 expression which allows more activin A to bind to its receptors and hence, enhances activin A-induced inhibin *β*_B_-subunit mRNA levels. Our hypothesis is further supported by results from two additional experimental approaches. First, reduced endogenous FST or FSTL3 mRNA and protein levels, after targeting siRNA transfection, augmented activin A-induced inhibin *β*_B_-subunit mRNA levels similar to those observed with GDF9 treatment (Figure 4B). Second, the enhancing effects of GDF9 on activin A-induced inhibin *β*_B_-subunit mRNA and inhibin B levels were attenuated by exogenous FST (both 288 and 315 isoforms) or FSTL3 in a dose-dependent manner (Figure 5).

Alternative precursor mRNA splicing produces two main isoforms of mature mammalian follistatin, a core protein of 315 amino acids (FST315) and a carboxy-truncated variant of 288 amino acids (FST288) [29]–[31]. Although both isoforms have a similar binding affinity for activin [32], FST288 also has a high affinity for heparin [32], [33]. In rat pituitary cells, complexes of activin and FST288 bind to cell surface proteoglycans via the heparin binding site of FST288 and is a mechanism by which activin is targeted for degradation [34]. Although FST315 has the same affinity for activin as FST288, it is primarily present in the human circulation [35] and does not bind to heparin. Instead, FST315 acts as a storage for follistatin in the circulation, which delivers activin to target cells and prevents activin from binding to FST288, and hence, degradation. Using TaqMan gene expression assay with probes that could distinguish these two FST isoforms (FST288 and FST315, Assay ID: Hs00246260_m1 and Hs01121164_m1, respectively, Applied Biosystems, Foster City, CA), we confirmed that GDF9 suppressed both isoforms in a time-dependent manner (See Experimental Procedures S1 and Figure S1, published on PLoS ONE online web site at http://www.plosone.org/). In addition, the basal mRNA levels of FST315 were about 10 fold higher than those of FST288 (Ct value of FST315 was about 3 cycles earlier than that of FST288). These results suggest that FST315 mRNA may play a more dominant role in our human granulosa-lutein cell culture system and are consistent with the overall trend of a greater suppression of inhibin *β*_B_ subunit and inhibin B levels by FST315 relative to that of FST288 at equivalent doses below the saturated dose of 50 ng/ml in our results (Figure 5). The relative ratio of activin-free to activin-bound FST is essential in determining the bioavailability of activin and estimating the potential endocrine function of circulating FST but free follistatin assays are not commercially available at present. Because FST binds activins and inhibins through the common *β*-subunit [24], we cannot exclude the possibility that FST may also interact with inhibins. However, FST has a 500 to 1000 fold higher affinity for activins than inhibins [36].

FSTL3, which shares several structural features with FST, does not have a heparin binding sequence and therefore, does not bind cell-surface proteoglycans under normal conditions [37]. Isoforms of FST are secreted faster than FSTL3 by stable, transfected CHO cells and the amount of newly synthesized FSTL3 localized in the nucleus is still substantial for up to 8 h, which is significantly longer than that for FST [38]. This may explain partly why the protein concentration of FST in the media of our cultured hGL cells was about eight fold higher than that of FSTL3 (Figure 1, E and F, 4450 *vs*. 548 pg/ml, *P*<0.001). Despite a similar decrease in FST or FSTL3 mRNA and protein levels 48 h after targeting siRNA transfection (Figure 4A), activin A-induced inhibin *β*_B_-subunit mRNA levels were higher following FST siRNA (Figure 4B; *P*<0.05). Furthermore, at the same dose of 4 ng/ml, FST288 or FST315 had a stronger effect than FSTL3 in suppressing activin A-induced inhibin *β*_B_-subunit mRNA in the presence of GDF9 although these differences did not reach statistical significance (Figure 5). Whether this indicates that FST may play a more dominant role than FSTL3 in regulating activin A action requires further studies.

FST protein levels were lower following FST siRNA transfection (from 4601 to 2544 pg/ml, Figure 5A, *lower* panel) than treatment with 100 ng/ml of GDF9 (from 4451 to 3266 pg/ml, Figure 1E), but corresponding activin A-induced inhibin *β*_B_-subunit mRNA and inhibin B levels were lower after FST siRNA transfection than GDF9 pre-treatment (Figure 4B). These quantitative differences in activin A-induced inhibin *β*_B_-subunit mRNA and inhibin B response may suggest that GDF9 acts through not just the extracellular mechanism mediated by FST, but as reported in our previous studies [16], [17], the intracellular mechanisms mediated by activin receptors and Smads. However, we cannot exclude the additional, although small, effect of FSTL3 accounting for this difference. In addition, we also cannot rule out the possibility that GDF9 may affect other extracellular inhibitors of activin such as the BMP and activin membrane-bound inhibitor (BAMBI). BAMBI is a transmembrane protein related to the TGF*β* superfamily type I receptors. BAMBI lacks an intracellular kinase domain but can block activin signaling by forming stable associations with activin type IB receptor (ACVR1B, also known as ALK4) but not activin type IA receptor (ACVR1A, also known as ALK2) [39].

Serum levels of total and activin-free FST were about 10.1±1.6 ng/ml and 1 ng/ml respectively in normal cycling women [40]. Thadhani *et al.* showed that women who developed gestational diabetes mellitus had lower first-trimester serum levels of FSTL3 compared with women who did not (median 10.789 *vs.* 30.670 ng/ml, *P* value <0.001) [41]. The FST concentrations used in our experiments did span the range observed under these physiological states. However, the clinical significance of our findings in normal physiology remains to be determined. In mouse models, over-expression of FST has been shown to result in a PCOS-like phenotype [42]. Higher FST levels observed in some women with polycystic ovary syndrome (PCOS) have led to the suggestion that altered FST function may contribute to the PCOS phenotype [43]; however, an updated study on allelic variants of the follistatin gene in PCOS suggests that the contribution of the follistatin gene to the etiology of PCOS is small [44]. Decreased GDF9 mRNA levels have been found in developing oocytes from women with PCOS or polycystic ovaries compared to women with normal ovaries; the decreased levels are evident throughout folliculogenesis, beginning at recruitment initiation and continuing through the small, Graafian follicle stage [45]. Although increased FST or FSTL3 expression with decreased endogenous GDF9 levels after targeting siRNA transfection (Figure 3) may provide a mechanism by which altered GDF9 expression can affect follicle development, our granulosa cells were not specifically obtained from women with PCOS. Future studies comparing the interactions of FST or FSTL3 and GDF9 in granulosa cells from women with and without PCOS may shed new insight on the pathophysiology of this condition.

We have recently reported that inhibin B, with its production enhanced by GDF9 pretreatment with activin A, can attenuate the inhibitory effect of activin A on steroidogenic acute regulatory protein [46] expression and progesterone production [47]. Activin A is a known inhibitor of luteinization as reported in human [48], [49], bovine [50], [51], and rat [52] ovaries. Serum free activin A levels show little variation in the human menstrual cycle [53] while serum inhibin B levels peak in the mid-follicular phase and immediately following ovulation [54]. Based on these observations and findings of our studies [16], [17], [47], we have previously speculated that GDF9 may enhance activin A-induced inhibin B production in the pre-ovulatory follicle sufficiently to overcome the inhibitory effect of activin A on luteinization. Our current study shows that GDF9 can also suppress mRNA and protein levels of FST and FSTL3, known inhibitors of activin A actions, and reverse their inhibitory effects on activin-induced inhibin B production. This further corroborates our proposal for a potential role of GDF9 in regulating activin A and inhibin B functions in human granulsa cells during the follicular-luteal transition [49].

While our granulosa cell culture systems had provided a convenient model to study the inter-relationships of FST or FSTL3 and GDF9, these cells were exposed to pharmacological doses of exogenous gonadotropins and were undergoing luteinization following hCG administration. However, in the absence of human granulosa cells from normal ovaries for research, findings from our cell culture systems do provide interesting hypotheses on the potential role of GDF9 in granulosa-lutein cell functions.

**Figure 6:**
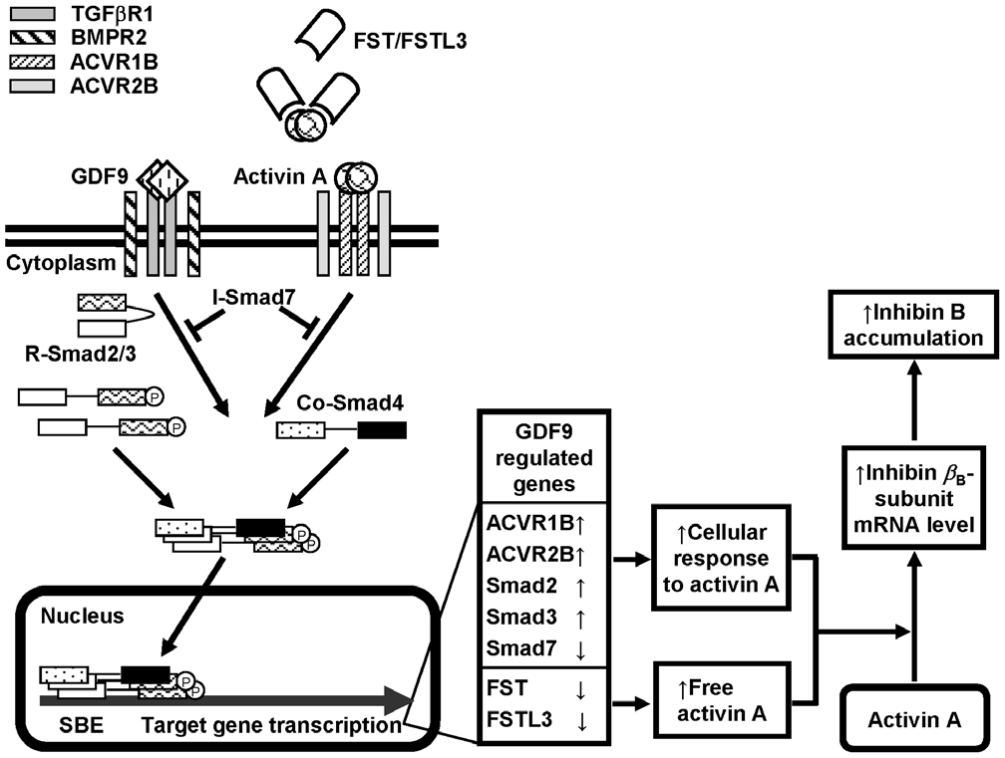
A model on the interactions between GDF9, activin A, and FST/FSTL3 in regulating inhibin B production. GDF9 increases hGL cell response to activin A by acting on GDF9 receptors, BMPR2/TGF*β*R1, which then activate Smad2/3 to form complexes with Smad4. These complexes then activate transcription factors in the nucleus to target genes that increase ACVR2B/1B and Smad2/3 expression and reduce Smad7 activity [16], [17]. These changes, in turn, allow more activin A to bind to its receptors and increase the cellular response to accumulate inhibin *β*_B_-subunit, and corresponding inhibin B intracellularly. Additionally, GDF9 inhibits FST and FSTL3 gene transcription and FST and FSTL3 secretion extracellularly, reducing the amount of FST and FSTL3 binding to activin A which then activates activin receptors and the downstream signaling cascade and increases inhibin B accumulation. Abbreviations: ACVR, activin receptor; BMPR2, bone morphogenetic protein type II receptor; FST, follistatin; FSTL3, follistatin-like 3; GDF9, growth differentiation factor 9; Smad, son of mothers against decapentaplegia; SBE, Smad binding element; Co-Smad, common Smad; I-Smad, inhibitory Smad; R-Smad, receptor-activated Smad; TGF*β*R1, transforming growth factor *β* type I receptor.

In summary, our previous studies suggest that GDF9 enhances activin A-induced inhibin *β*_B_-subunit mRNA levels in hGL cells by regulating receptors and crucial intracellular components of the activin signaling pathway involving the Smad system [16], [17]. Our current study shows that GDF9 can decrease FST and FSTL3 expression in addition, which then allows more free activin A to bind to its receptors and activate the signaling pathway downstream. Taken together and as summarized in Figure 6, GDF9 increases hGL cell response to activin A by acting on GDF9 receptors, BMPR2/TGF*β*R1, which then activate Smad2/3 to form complexes with Smad4. These complexes then activate transcription factors in the nucleus to target genes that increase ACVR2B/1B and Smad2/3 expression and reduce Smad7 activity. These changes, in turn, allow more activin A to bind to its receptors and increase the cellular response to accumulate inhibin *β*_B_-subunit, and corresponding inhibin B levels intracellularly. Additionally, GDF9 can inhibit FST and FSTL3 secretion extracellularly hence, the amount of activin A binding to FST and FSTL3. More activin A can then interact with its receptors to activate the downstream signaling cascade, leading to increased inhibin B accumulation.

Although we have described the intracellular and extracellular mechanisms as separate GDF9 actions, whether the extracellular mechanism alone is sufficient to explain for the changes in the intracellular pathway or whether GDF9 also has a separate intracellular regulating role needs further research. Further investigations will also be required to characterize the mechanism on how GDF9 suppresses FST and FSTL3 expression, and whether the Smad pathway is directly involved.

## Supporting Information

Figure S1 **Time-dependent effect of GDF9 on FST288 and FST315 mRNA levels.** After 48 h preculture, the culture media were replaced with low-serum media (0.5% FBS); hGL cells were then treated with 100 ng/ml recombinant human GDF9 for 12 h (“Time 12 h”), 24 h (“Time 24 h”) and 48 h (“Time 48 h”) in time-dependent experiments. FST288 and FST315 mRNA levels in hGL cells were assessed by TaqMan Gene Expression Assay. Results were the means ± SEM from at least three sets of experiments (each from a separate patient), and in each set, measurements were made in triplicate. *Means without a common letter* are significantly different (*P*<0.05).
(TIF)

Experimental Procedures S1 **TaqMan Gene Expression Assays.**
(DOC)
