# Supplementary figures and images for: Growth Differentiation Factor 9 (GDF9) Suppresses Follistatin and Follistatin-Like 3 Production in Human Granulosa-Lutein Cells
Source: PLoS One. 2011 Aug 1;6(8):e22866. doi: 10.1371/journal.pone.0022866 (PMC3148233; doi:10.1371/journal.pone.0022866)

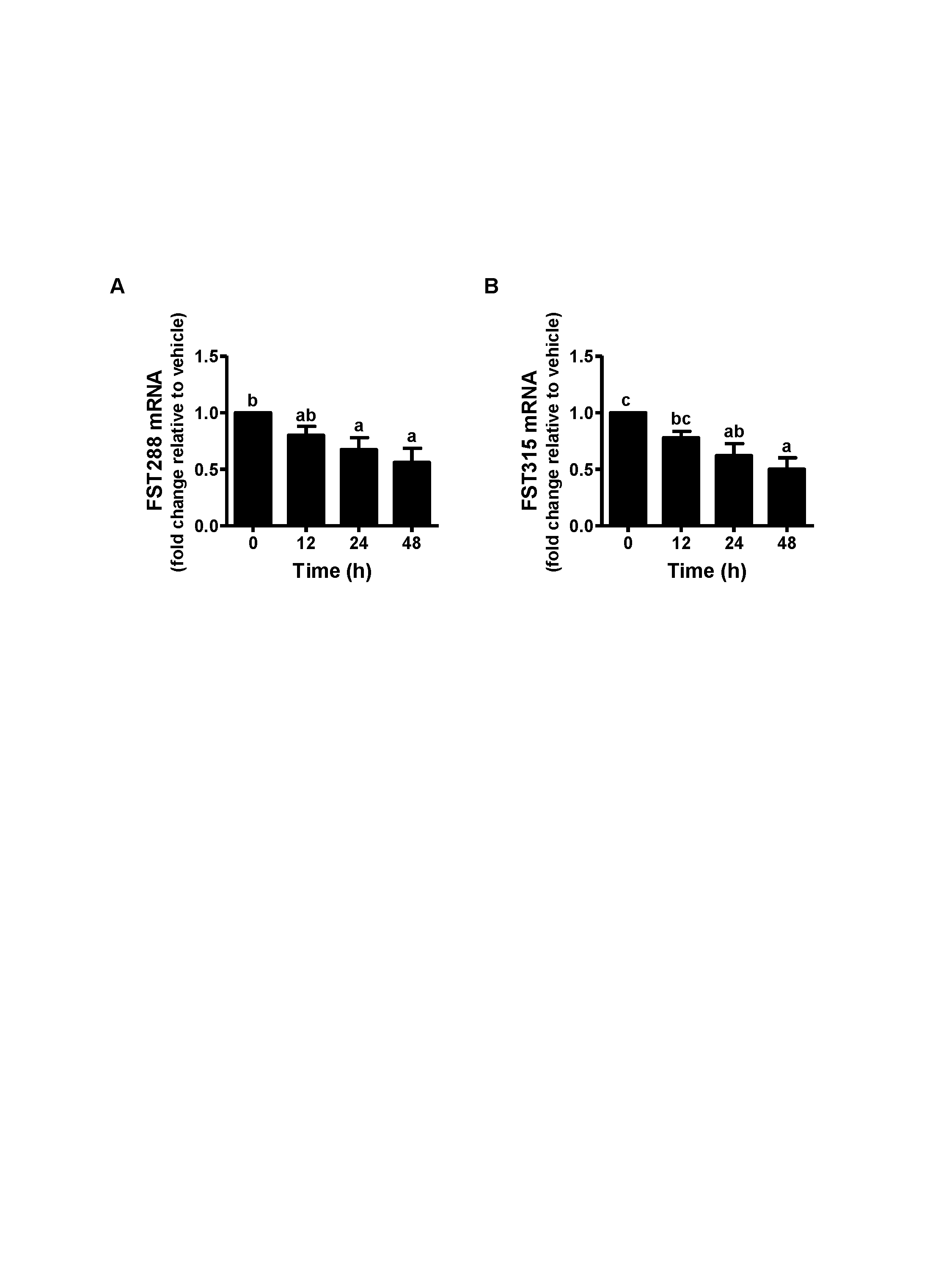

Supplement: Figure S1 — Time-dependent effect of GDF9 on FST288 and FST315 mRNA levels. After 48 h preculture, the culture media were replaced with low-serum media (0.5% FBS); hGL cells were then treated with 100 ng/ml recombinant human GDF9 for 12 h (“Time 12 h”), 24 h (“Time 24 h”) and 48 h (“Time 48 h”) in time-dependent experiments. FST288 and FST315 mRNA levels in hGL cells were assessed by TaqMan Gene Expression Assay. Results were the means ± SEM from at least three sets of experiments (each from a separate patient), and in each set, measurements were made in triplicate. Means without a common letter are significantly different (P<0.05). (TIF) [file pone.0022866.s001.tif]
