# Supplementary material for: Growth Differentiation Factor 9 (GDF9) Suppresses Follistatin and Follistatin-Like 3 Production in Human Granulosa-Lutein Cells
Source: PLoS One. 2011 Aug 1;6(8):e22866. doi: 10.1371/journal.pone.0022866 (PMC3148233; doi:10.1371/journal.pone.0022866)
Supplement: Experimental Procedures S1 — TaqMan Gene Expression Assays. (DOC) [file pone.0022866.s002.doc]

**Experimental Procedure S1**

*TaqMan Gene Expression Assays*-cDNAs used for TaqMan Gene Expression Assays were same as those used for SYBR Green real-time PCR. The primers and probes used for TaqMan Gene Expression Assays were as following: human FST288, Hs00246260_m1 and human FST315, Hs01121164_m1 (Applied Biosystems). For TaqMan Gene Expression Assays, 10 ng of cDNA were mixed with gene-specific TaqMan primer/probe and TaqMan Gene Expression Master Mix (4370048; Applied Biosystems). Real-time PCR were performed on the ABI PRISM® 7300 Sequence Detection System according to the manufacturer's protocol (Applied Biosystems). Amplification specificity using the melting curve and analysis and quantification of the relative mRNA levels using the comparative Ct method were carried out on the ABI Prism 7300 Sequence Detection Software version 1.3 (Applied Biosystems). Expression levels were quantified and normalized to those of the human housekeeping gene, GAPDH (hCG2005673, Applied Biosystems). Changes after treatments were recorded as fold differences from values in untreated controls at each time point as appropriate.
